# Assessment of left atrioventricular coupling and left atrial function impairment in diabetes with and without hypertension using CMR feature tracking

**DOI:** 10.1186/s12933-023-01997-z

**Published:** 2023-10-30

**Authors:** Rui Shi, Yi-Ning Jiang, Wen-Lei Qian, Ying-Kun Guo, Yue Gao, Li-Ting Shen, Li Jiang, Xue-Ming Li, Zhi-Gang Yang, Yuan Li

**Affiliations:** 1 Department of Radiology, West China Hospital, Sichuan University, No. 37 Guo Xue Xiang, Chengdu, 610041 Sichuan China; 2 Department of Radiology, Key Laboratory of Birth Defects and Related Diseases of Women and Children of Ministry of Education, West China Second University Hospital, Sichuan University, Chengdu, China

**Keywords:** Diabetes mellitus, Hypertension, Left atrial dysfunction, Cardiomyopathy, Cardiac MRI

## Abstract

**Purpose:**

The study was designed to assess the effect of co-occurrence of diabetes mellitus (DM) and hypertension on the deterioration of left atrioventricular coupling index (LACI) and left atrial (LA) function in comparison to individuals suffering from DM only.

**Methods:**

From December 2015 to June 2022, we consecutively recruited patients with clinically diagnosed DM who underwent cardiac magnetic resonance (CMR) at our hospital. The study comprised a total of 176 patients with DM, who were divided into two groups based on their blood pressure status: 103 with hypertension (DM + HP) and 73 without hypertension (DM-HP). LA reservoir function (reservoir strain (ε_s_), total LA ejection fraction (LAEF)), conduit function (conduit strain (ε_e_), passive LAEF), booster-pump function (booster strain (ε_a_) and active LAEF), LA volume index (LAVI), LV global longitudinal strain (LVGLS), and LACI were evaluated and compared between the two groups.

**Results:**

After adjusting for age, sex, body surface area (BSA), and history of current smoking, total LAEF (61.16 ± 14.04 vs. 56.05 ± 12.72, p = 0.013) and active LAEF (43.98 ± 14.33 vs. 38.72 ± 13.51, p = 0.017) were lower, while passive LAEF (33.22 ± 14.11 vs. 31.28 ± 15.01, p = 0.807) remained unchanged in the DM + HP group compared to the DM-HP group. The DM + HP group had decreased ε_s_ (41.27 ± 18.89 vs. 33.41 ± 13.94, p = 0.006), ε_e_ (23.69 ± 12.96 vs. 18.90 ± 9.90, p = 0.037), ε_a_ (17.83 ± 8.09 vs. 14.93 ± 6.63, p = 0.019), and increased LACI (17.40±10.28 vs. 22.72±15.01, p = 0.049) when compared to the DM-HP group. In patients with DM, multivariate analysis revealed significant independent associations between LV GLS and εs (β=-1.286, p < 0.001), εe (β=-0.919, p < 0.001), and εa (β=-0.324, p = 0.036). However, there was no significant association observed between LV GLS and LACI (β=-0.003, p = 0.075). Additionally, hypertension was found to independently contribute to decreased εa (β=-2.508, p = 0.027) and increased LACI in individuals with DM (β = 0.05, p = 0.011).

**Conclusions:**

In DM patients, LV GLS showed a significant association with LA phasic strain. Hypertension was found to exacerbate the decline in LA booster strain and increase LACI in DM patients, indicating potential atrioventricular coupling index alterations.

## Introduction

Cardiovascular disease is the most common cause of mortality of diabetes mellitus (DM) patients. Due to overlapping pathophysiological factors, hypertension and DM often coexist as lifestyle diseases. Approximately 50% of individuals with type 2 DM also suffer from hypertension, with even higher rates observed among hospitalized patients [1]. These conditions can induce structural remodeling and cardiac dysfunction. The DM and hypertension-related cardiomyopathy is defined as the pathological changes in the left ventricular (LV) myocardium, beginning with diastolic dysfunction with normal systolic function [2, 3]. As the condition progresses, there may be a potential for the development of LV systolic dysfunction, which can be associated with heart failure with reduced ejection fraction and, in severe cases, could have serious outcomes, including mortality [4]. However, the left atrial (LA) is vital in regulating LV function [5]. LA’s function has three phases: reservoir, conduit, and pump [6]. LA dysfunction can result in diminished cardiac performance, even in the presence of preserved LV systolic function in DM patients [7, 8]. The left atrioventricular coupling index (LACI) is a newly identified marker with a strong association to adverse cardiovascular outcomes [9, 10].

The noninvasive imaging techniques have sparked interest in studying functional changes in the LA across various diseases. Echocardiography, being the most convenient method to evaluate LA size and function, faces limitations such as the acoustic window, complex LA structure, and thin atrial wall [11]. One of the significant benefits of CMR is its scanning window, which remains largely unaffected by air-filled pulmonary tissue. CMR can provide more consistent and reliable images of cardiac structures and function, particularly in patients with suboptimal acoustic windows due to pulmonary conditions or other anatomical factors [12, 13].

The deterioration of LV function by DM and/or hypertension has been demonstrated [14–16], but it is uncertain whether DM with hypertension leads to further deterioration of LA function and LACI. The purpose of this study was to determine the effect of hypertension on LA structural and functional changes in individuals with DM, utilizing MRI-based feature tracking, and to study the interaction between atrial and ventricular volumes and function using LACI as measure in these individuals.

## Methods and materials

### Study population

This study received institutional review board approval and informed consent was waived due to its retrospective design. From December 2015 to June 2022, we consecutively recruited 607 patients with clinically diagnosed T2DM who underwent cardiac MR at our hospital. T2DM diagnosis based on American Diabetes Association guidelines or treatment with glucose-lowering pharmacotherapy [17]. Exclusion criteria were: primary cardiomyopathies (hypertrophic cardiomyopathy (n = 27), restrictive cardiomyopathy (n = 19), and dilated cardiomyopathy (n = 62)), individuals with ischemic cardiomyopathy (n = 97), severe valvular heart disease (n = 32), congenital heart disease (n = 17), Incomplete clinical data (n = 25), severe cardiac arrhythmia (n = 43), inadequate image quality (n = 27), incomplete imaging analysis results(n = 11), and CMR derived LVEF < 50% (n = 71).

Finally, 176 consecutive DM patients were enrolled and divided into two groups: DM-HP (n = 73) and DM + HP (n = 103), based on the presence or absence of concomitant hypertension. Hypertension was diagnosed based on either antihypertensive treatment use or clinically diagnosed hypertension (systolic BP ≥ 140 mmHg and/or diastolic BP ≥ 90 mmHg, taking on at least two occasions). Patient data on clinical variables, family history, medication use, and other risk factors was collected through medical records.

### MR protocols

Cardiac MR scans were performed using 3.0 T Siemens MAGNETOM Trio Tim or Skyra scanners (Siemens Medical Solutions, Germany). Cine images were acquired using balanced steady-state free precession in standard short- and long-axis views during end-expiratory breath-hold. Parameters for the Trio Tim system were: TR = 40.35ms, TE/TR = 3.4/1.3ms, matrix = 208 × 139, flip angle = 50°, FOV = 250 × 300 mm, slice thickness = 8 mm (with a 2 mm gap between the slices), 25 frames per cycle. Parameters for the Skyra system were: TR = 39.34ms, TE/TR = 2.81/1.22ms, segment = 14, flip angle = 38°, slice thickness = 8 mm (with a 2 mm gap between the slices), FOV = 360 × 300 mm, matrix = 256 × 166, 25 frames per cycle.

### MR analysis

#### LA and LV structural and functional analysis

**Fig. 1:**
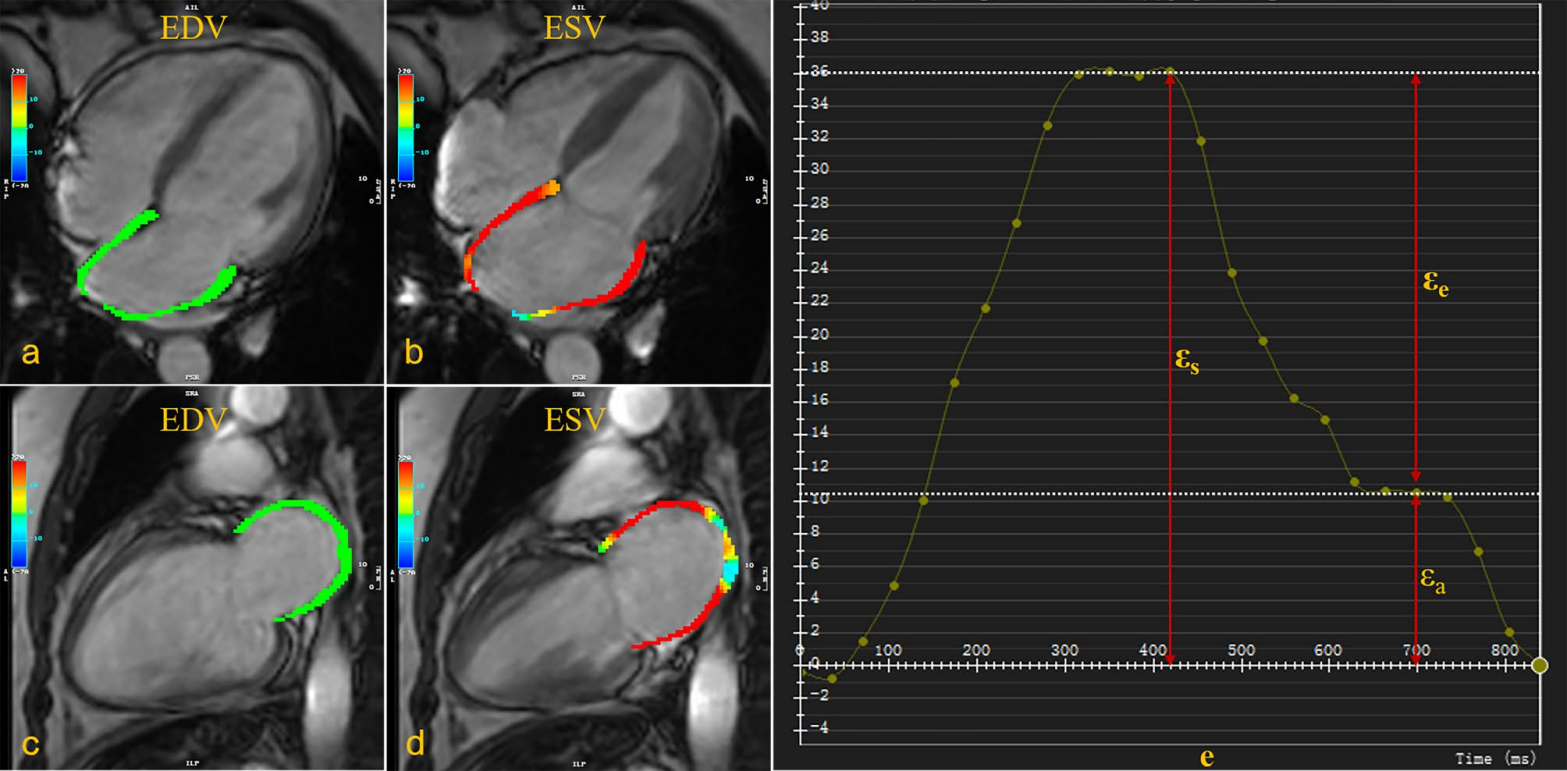
Representative Images and Plot of MRI-Derived Left Atrial Longitudinal Strain. **(a)** Four-chamber view of left atrial longitudinal strain at end-diastolic phase; **(b)** Four-chamber view of left atrial longitudinal strain at end-systolic phase; **(c)** Two-chamber view of left atrial longitudinal strain at end-diastolic phase. **(d)** Two-chamber view of left atrial longitudinal strain at end-systolic phase; **(e)** Plot of left atrial phasic longitudinal strain, including reservoir strain (ε_s_), conduit strain (ε_e_), and booster strain (ε_a_)

Two experienced radiologists (S.R and Q.W.L with 3–4 years of experience in cardiac MR analysis) quantified LA and LV myocardial deformation using the commercial software cvi42 (Circle Cardiovascular Imaging, Calgary, Canada). LV function parameters, including myocardial mass (LVM), LV end-diastolic volume (LVEDV), LV end-systolic volume (LVESV), and LV ejection fraction (LVEF), were calculated by tracing contiguous short-axis cine images from the apex to the mitral valve plane. Papillary muscles were included within the LV volume. The LVM index (LVMI), LVEDV index (LVEDVI), and LVESV index (LVESVI) were standardized using body surface area (BSA). The LV remodeling index (LVRI) was determined by calculating the ratio of LVM to LVEDV. LA myocardial strain was analyzed by outlining the LA borders excluding the LA appendage and pulmonary veins in apical 2- and 4-chamber views. LV myocardial strain was traced in the same manner in short axis, 2- and 4-chamber views. The LV end-diastole was used as the reference point. An automated tracking algorithm was applied with manual adjustments for optimal wall tracking. The software automatically derived LV global longitudinal strain (LV GLS) and LA reservoir (ε_s_), conduit (ε_e_), and booster (ε_a_) strains (Fig. 1).

**Fig. 2:**
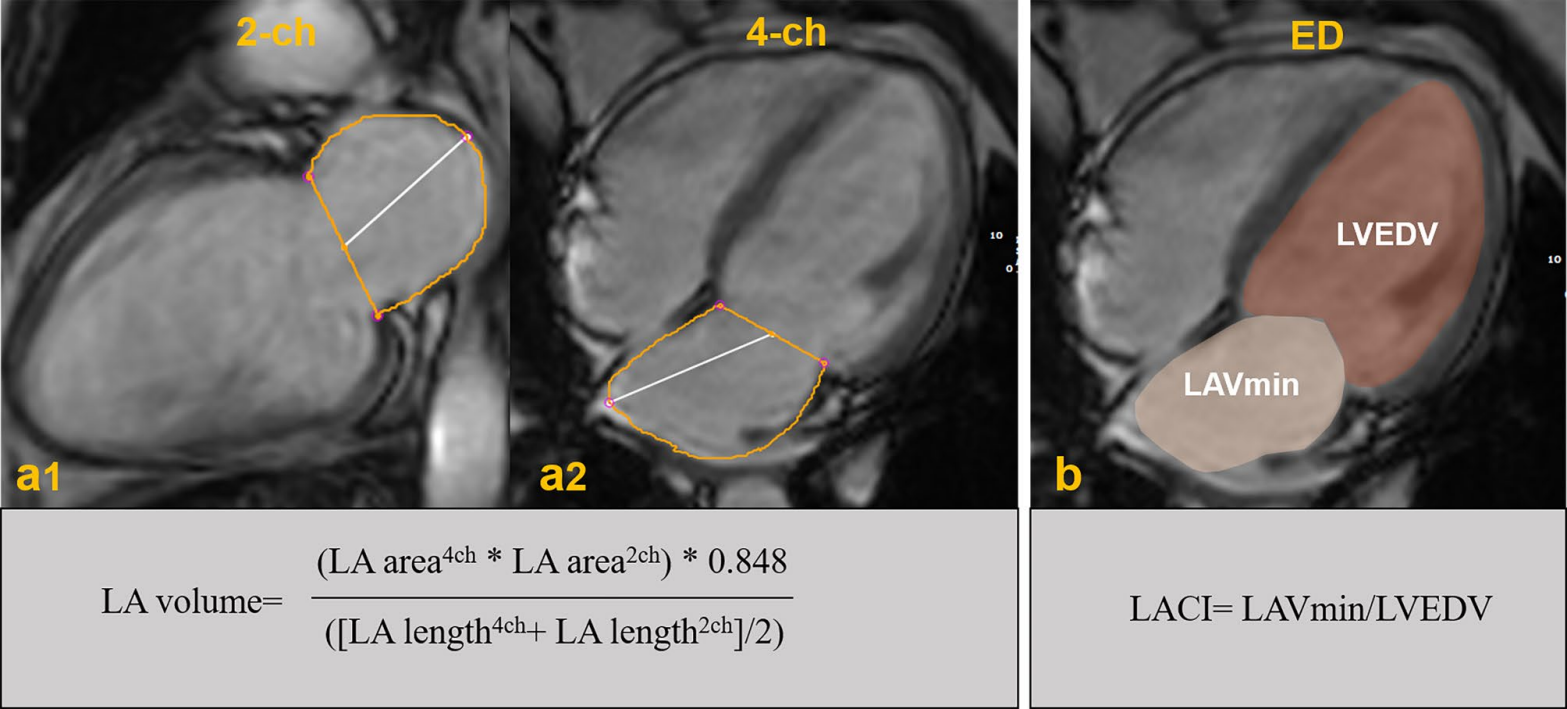
A schematic representation of the left atrial volume and left atrioventricular coupling index (LACI). The left atrial area for the 2-chamber **(a1)** and 4-chamber **(a2)** views. The left atrial volume was calculated using the biplane formula mentioned above. The left atrial volume and left ventricular volume at end-diastole are represented in **(b)**. The LACI was obtained by calculating the ratio of end-diastolic left atrial volume to left ventricular volume

LA volumetric function analysis was performed with the same commercially available software (cvi42, Circle Cardiovascular Imaging, Calgary, Canada). The schematic representation for LA and ventricular volumes, along with associated formulas is shown in Fig. 2. The LA volume (LAV) was computed using the previously described formula [18]. The assessment of LAV was carried out at three crucial phases: end-systole (LAVmax), pre-contraction diastole (LAVpre), and late end-diastole post-contraction (LAVmin). Phasic LAV was normalized based on body surface area (BSA) resulting in LAVmax index (LAVImax), LAVpre index (LAVIpre), and LAVmin index (LAVImin). The calculation of LA total ejection fraction (total LAEF), passive ejection fraction (passive LAEF) was based on these phases. The LACI was determined by computing the ratio of LAVmin to LVEDV.

### Reproducibility analysis

To evaluate intra- and inter-observer variability, LV GLS, LA strain, and phasic LAV were measured in 60 subjects (30 per group) by a radiologist (S.R, with 4 years of experience in cardiac MRI diagnosis). The measurements were repeated after a 2-week interval. Additional investigators (Q.W.L, with 3 years of experience in cardiac MRI diagnosis) independently reanalyzed the data, without access to the initial contours and analysis results. Intra-observer variability was assessed using the results of the first investigator’s two measurements. The results of all two investigators were used to assess inter-observer variability.

### Statistics

The presentation of categorical data in this study is given as frequency (%). Group comparisons were conducted utilizing the chi-squared test, except in cases where the expected frequency was less than 5, where the Fisher’s exact test was employed. Continuous variables were represented as mean ± standard deviation (SD) for normally distributed data or as median (interquartile range) for non-normally distributed data. Group comparisons for continuous variables were made using either an independent t-test or the Mann-Whitney U test. Differences in CMR-derived parameters between DM-HP and DM + HP were compared by analysis of covariance after adjusting for age, sex, BSA, and smoking history. The correlation coefficient between CMR-derived parameters in DM patients were evaluated using the spearman rank correlation.

Multivariable linear regression was used to evaluate the associations between clinical factors and LA phasic strain and LACI, respectively. Clinical confounding factors and variables that were significant (p < 0.05) in the univariate analysis were included in the multivariable model. To prevent multicollinearity among the univariate variables, a variance inflation factor of 5 was applied. The LA VImax, LA VIpre, and LA VImin demonstrated covariance, and LAVImax was added to the model given its established impact on adverse prognosis. Intra- and inter-observer variability of LA phasic strain, LV GLS, and phasic LAV was evaluated using Interclass Correlation Coefficients (ICCs). The statistical analysis was carried out using IBM SPSS Statistics version 25.0 (IBMCorp., Armonk, NY, USA). A two-tailed P-value of less than 0.05 was considered statistically significant.

## Results

### Clinical characteristics of the diabetic population included in the study

The demographic characteristics, including age (57±11 vs. 61±11, p = 0.075) and sex (male, 53% vs. 58%, p = 0.623), were similar between the DM-HP and DM + HP groups. The DM + HP group had a larger BSA (1.65±0.15 vs. 1.70±0.20, p = 0.008) and higher systolic blood pressure (SBP, 125±14 vs. 134±17, p < 0.001) compared to the DM-HP group. A higher proportion of current smoking history were found in the DM + HP group compared to DM-HP group (17.8% vs. 33.9%, p = 0.022). Total cholesterol levels were lower in the DM + HP group compared to the DM-HP group (4.49±1.40 vs. 4.05±1.20, p = 0.027). A summary of the demographic and clinical characteristics of these subjects can be found in Table 1.

**Table 1:** Baseline Demographic and Clinical Features of the Study Cohort

	DM-HP (n = 73)	DM + HP (n = 103)	P value
Demographics			
sex, male, n(%)	39(53)	60(58)	0.623
Age, years	57±11	61±11	0.075
BSA, m^2^	1.65±0.15	1.70±0.20	0.008
Dyslipidemia, n(%)	27(37.0)	42(40.8)	0.716
Current smoking, n(%)	13(17.8)	35(33.9)	0.022
SBP, mmHg	125±14	134±17	< 0.001
DBP, mmHg	78±12	80±12	0.356
**Laboratory indices**			
HbA1c (%)	7.34±1.70	7.00±1.22	0.179
TG, mmol/l	2.00±1.77	1.62±1.09	0.075
TC, mmol/l	4.49±1.40	4.05±1.20	0.027
HDL-C, mmol/l	1.21 + 0.34	1.23±0.38	0.718
LDL-C, mmol/l	2.60±1.47	2.31±1.02	0.118
**Medications, n (%)**			
ACEI/ARB	12(16.4)	87(84.5)	< 0.001
Beta-blocker	4(5.5)	13(12.6)	< 0.001
Calcium channel blocker	3(4.1)	27(26.2)	< 0.001
Diuretics	5(6.8)	25(24.3)	0.005
**anti-diabetic treatment, n (%)**			
insulin	11(15.1)	18(17.5)	0.671
metformin	12(16.4)	21(20.4)	0.508
sulfonylurea	18(24.7)	27(26.3)	0.816
α-glucosidase inhibitor	20(27.4)	24(23.3)	0.536
other	2(2.7)	4(3.9)	0.479
non-drug	13(17.8)	16(15.6)	0.689

Note: Data are presented as mean±SD or number (percentage)

Abbreviations: DM-HP: diabetes without hypertension; DM + HP: diabetes with hypertension; BSA: body surface area; SBP: systolic blood pressure; DBP: diastolic blood pressure; TG: triglycerides; TC: total cholesterol; HDL: high-density lipoprotein; LDL: low-density lipoprotein; ACEI/ARB: Angiotensin Converting Enzyme Inhibitor/ Angiotensin Receptor Blocker

### Comparison of the characteristics of the cardiac MRI-derived parameters between DM-HP and DM + HP group

Cardiac MRI parameters are presented in Table 2. After adjusting for age, sex, body surface area (BSA), and history of current smoking, no significant differences were observed in LVEDVI, LVESVI, and LVEF between the DM-HP and DM + HP groups (all p > 0.05). The DM + HP group demonstrated increased LVMI (45.40 ± 13.74 vs. 53.95 ± 14.63, p < 0.001) and LVRI (0.61 ± 0.19 vs. 0.70 ± 0.20, p = 0.005) and decreased LV GLS (-12.67 ± 3.81 vs. -11.31 ± 3.68, p = 0.026) when compared to the DM-HP group.

**Table 2:** Comparison of CMR-Derived Parameters in DM-HP and DM + HP Groups Using Analysis of Covariance

		DM-HP (n = 73)	DM + HP (n = 103)	p-value#
LA parameters				
LAVImax(ml/m^2^)		32.71±12.32	39.02±15.99	0.005
LAVIpre(ml/m^2^)		22.53±10.45	27.06±14.65	0.025
LAVImin(ml/m^2^)		13.05±8.57	18.51±13.84	0.003
Total LAEF (%)		61.16±14.04	56.05±12.72	0.013
Passive LAEF (%)		33.22±14.11	31.28±15.01	0.807
Active LAEF (%)		43.98±14.33	38.72±13.51	0.017
ε_s_ (%)		41.27±18.89	33.41±13.94	0.006
ε_e_ (%)		23.69±12.96	18.90±9.90	0.037
ε_a_ (%)		17.83±8.09	14.93±6.63	0.019
**LV parameters**				
LVEDVI(ml/m^2^)		75.95±16.72	79.80±19.84	0.105
LVESVI(ml/m^2^)		29.66±8.29	30.62±10.37	0.384
LVEF(%)		60.72±6.23	61.58±6.21	0.327
LVMI(g/m^2^)		45.40±13.74	53.95±14.63	< 0.001
LVRI(g/ml)		0.61±0.19	0.70±0.20	0.005
LV GLS (%)		-12.67±3.81	-11.31±3.68	0.026
**LACI(%)**		17.40±10.28	22.72±15.01	0.049

**Note**: Data are presented as the mean ± SD. #: adjusted for age, sex, BSA, and smoking

**Abbreviations**: LAVImax: maximum left atrial volume index; LAVImin: minimum left atrial volume index; LAVIpre: left atrial volume index just before left atrial contraction; LAEF: left atrial emptying fraction; *ε*_*s*_, reservoir strain; *ε*_*e*_, conduit strain; *ε*_*a*_, booster strain; LVEDVI, left ventricular end-diastolic volume index; LVESVI, left ventricular end-systolic volume index; LVMI, left ventricular mass index; LVRI, left ventricular mass to end-diastolic ratio; LV GLS, left ventricular global peak longitudinal strain. LACI: left atrioventricular coupling index

**Fig. 3:**
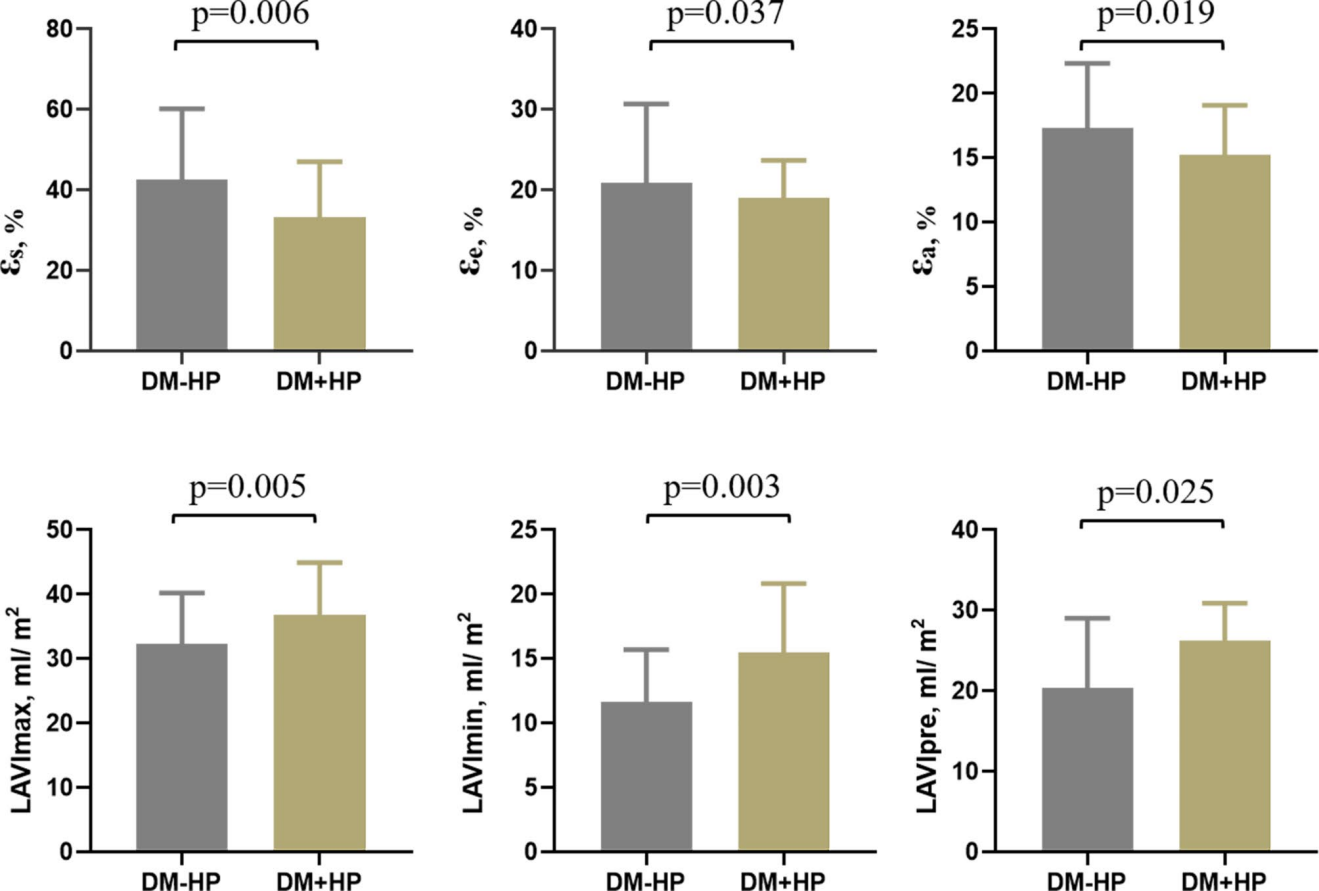
Comparison of left atrial phasic strain and volume index between DM-HP and DM + HP groups. Note: εs, reservoir strain; εe, conduit strain; εa, booster strain; LAVImax: maximum left atrial volume index; LAVImin: minimum left atrial volume index; LAVIpre: left atrial volume index just before left atrial contraction

LAVImax (32.71±12.32 vs. 39.02±15.99, p = 0.005), LAVIpre (22.53±10.45 vs. 27.06±14.65, p = 0.025), and LAVImin (13.05±8.57 vs. 18.51±13.84, p = 0.003) were increased in DM + HP group than DM-HP group. The DM + HP group had decreased ε_s_ (41.27 ± 18.89 vs. 33.41 ± 13.94, p = 0.006), ε_e_ (23.69 ± 12.96 vs. 18.90 ± 9.90, p = 0.037), ε_a_ (17.83 ± 8.09 vs. 14.93 ± 6.63, p = 0.019) when compared to the DM-HP group (Fig. 3). Total LAEF (61.16 ± 14.04 vs. 56.05 ± 12.72, p = 0.013) and active LAEF (43.98 ± 14.33 vs. 38.72 ± 13.51, p = 0.017) were lower, while passive LAEF (33.22 ± 14.11 vs. 31.28 ± 15.01, p = 0.807) remained in the DM + HP group compared to the DM-HP group. The DM + HP group had increased LACI (17.40±10.28 vs. 22.72±15.01, p = 0.049) when compared to the DM-HP group.

### Correlation analysis of cardiac MRI parameters among DM patients

**Fig. 4:**
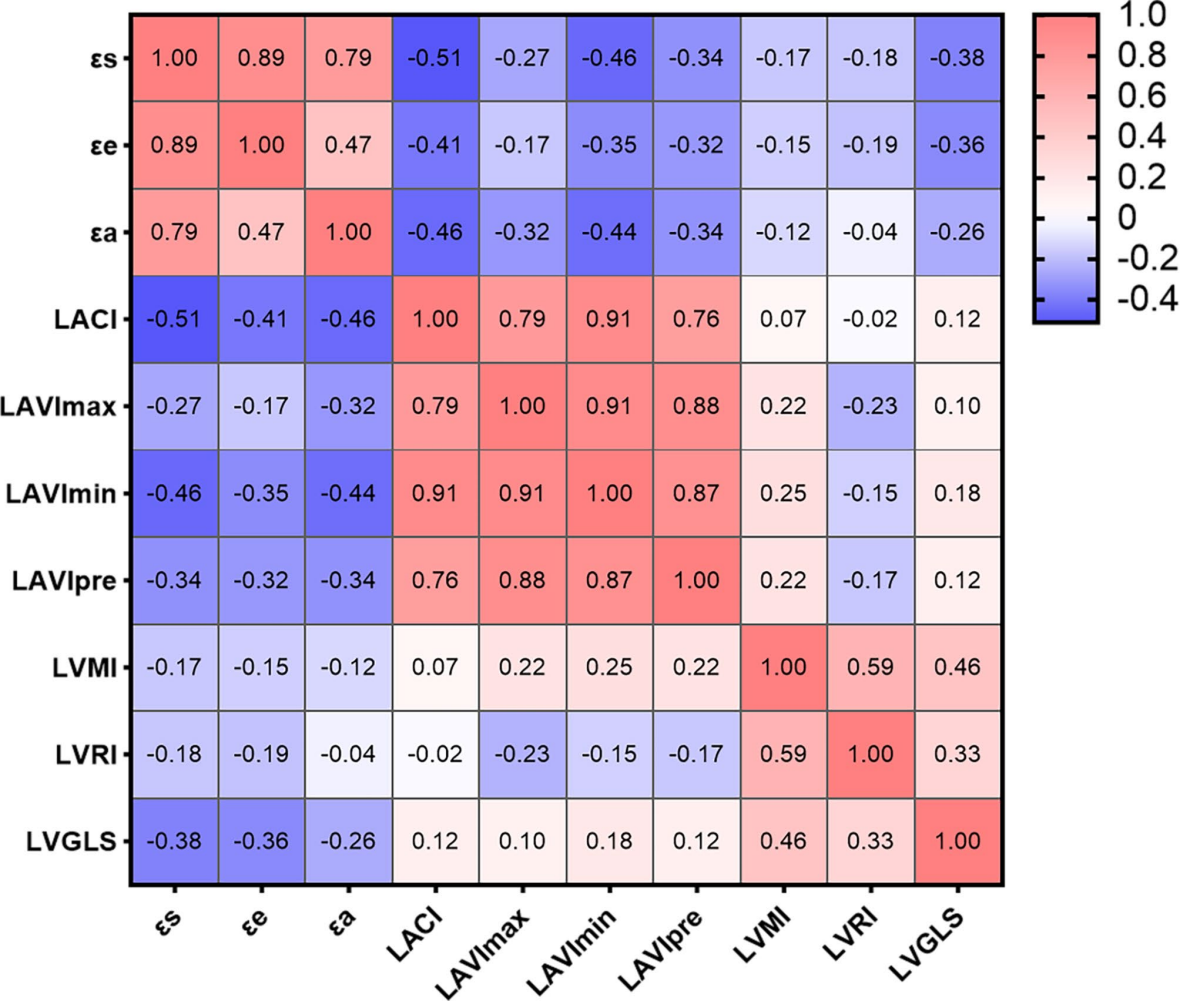
Heat maps illustrating the relationships between LA and LV parameters derived from CMR in patients with DM. The color shading of each cell corresponds to the magnitude and direction of the correlation coefficient, with red indicating a positive correlation and blue indicating a negative correlation. The numerical values within the cells represent the bivariate correlation coefficient. The abbreviations are consistent with Table 2

Correlation analysis of cardiac MRI-derived LA and LV parameters is presented as a heat map in Fig. 4. Significant correlations were found between εs, εe, and εa with GLS (εs: r=-0.383, εe: r=-0.360, εa: r=-0.258, all p < 0.05), LA VmaxI (εs: r=-0.296, εe: r=-0.202, εa: r=-0.327, all p < 0.05), LA VpreI (εs: r=-0.337, εe: r=-0.317, εa: r=-0.336, all p < 0.001), and LA VminI (εs: r=-0.460, εe: r=-0.354, εa: r=-0.445, all p < 0.001). εs and εe were slightly associated with LVMI (εs: r=-0.170, p = 0.024; εe: r=-0.148, p = 0.049) and LVRI (εs: r=-0.177, p = 0.019; εe: r=-0.189, p = 0.012).

### Univariate and multivariate regression analyses of LA strain and LACI in patients with DM

Table 3 displays the outcomes of both univariate and multivariate analyses conducted on LA phasic strain and LACI in patients with DM. The univariate regression analysis showed that hypertension was associated with ε_s_ (β=-9.357, p < 0.001), ε_e_ (β=-5.286, p = 0.002), ε_a_ (β=-3.642, p = 0.001), and LACI (β = 0.065, p = 0.001) in patients with DM. Multivariate analysis showed that hypertension was only associated with ε_a_ (β=-2.608, p = 0.027) and LACI (β = 0.05, p < 0.011) in these patients.

**Table 3:** Univariate and Multivariate Analysis of Left Atrial Phasic Strain and LACI in Patients with Diabetes

	εs (%)						εe (%)						εa (%)						LACI				
	Univariable			multivariable			Univariable			multivariable			Univariable			multivariable			Univariable			multivariable	
	β	P		β	P		β	P		β	P		β	P		β	p		β	p		β	p
**Age**	-0.546	< 0.001		-0.417	< 0.001		-0.463	< 0.001		-0.432	< 0.001		-0.085	0.086					0.005	< 0.001		0.002	< 0.001
**Sex**	-2.729	0.267					-3.332	0.053					0.345	0.758					-0.053	0.010			
**BSA**	-7.533	0.269					-7.848	0.100					0.170	0.957					-0.088	0.122			
**HP**	-9.357	< 0.001					-5.286	0.002					-3.642	0.001		-2.508	0.027		0.065	0.001		0.05	0.011
**Smoking**	-5.329	0.051					-5.135	0.007		-3.812	0.033		-0.111	0.929					-0.020	0.390			
**Dyslipidemia**	-4.651	0.064					-3.275	0.063					-1.864	0.100					-0.004	0.846			
**LVMI**	-0.216	0.009					-0.151	0.009					-0.061	0.105					0.000	0.806			
**LV GLS**	-1.608	< 0.001		-1.286	< 0.001		-1.133	< 0.001		-0.919	< 0.001		-0.436	0.003		-0.324	0.036		0.006	0.030		0.003	0.075
**LAVImax**	-0.422	< 0.001		-0.304	< 0.001		-0.206	< 0.001					-0.205	< 0.001		-0.194	< 0.001		0.008	< 0.001		0.007	< 0.001

**Abbreviations**: *ε*_*s*_, reservoir strain; *ε*_*e*_, conduit strain; *ε*_*a*_, booster strain; HP: hypertension; BSA: body surface area; LVMI, left ventricular mass index; LV GLS, left ventricular global peak longitudinal strain; LAVImax: maximum left atrial volume index

### Reproducibility analysis

The intra- and inter-observer variability of LA phasic strain, LV GLS, and phasic LAV measurement using MRI are presented in the Table 4. The intra-observer ICCs for LA phasic strain range from 0.959 to 0.973, while the inter-observer ICCs range from 0.804 to 0.899. For LVGLS, the intra-observer ICCs was 0.958, and the inter-observer ICCs was 0.875. The intra-observer ICCs for phasic LAV range from 0.964 to 0.993, and the inter-observer ICCs range from 0.904 to 0.980.

**Table 4:** Inter- and intra-observer variability of LA and LV strain

	intra-observer ICC (95%CI)	P value	inter-observer ICC (95%CI)	P value
**LA strain**				
*ε*_*s*_	0.973(0.956,0.983)	< 0.001	0.899(0.845,0.935)	< 0.001
*ε*_*e*_	0.970(0.953,0.981)	< 0.001	0.874(0.806,0.918)	< 0.001
*ε*_*a*_	0.959(0.935,0.974)	< 0.001	0.804(0.702,0.873)	< 0.001
**LV GLS**	0.958(0.935,0.974)	< 0.001	0.875(0.809,0.919)	< 0.001
**LA volume**				
LAVmax	0.964(0.922,0.982)	< 0.001	0.957(0.921,0.977)	< 0.001
LAVpre	0.965(0.935,0.981)	< 0.001	0.904(0.826,0.948)	< 0.001
LAVmin	0.993(0.987,0.996)	< 0.001	0.980(0.941,0.991)	< 0.001

**Abbreviations**: *ε*_*s*_, reservoir strain; *ε*_*e*_, conduit strain; *ε*_*a*_, booster strain; LV GLS, left ventricular global peak longitudinal strain;LAVmax: maximum left atrial volume index; LAVmin: minimum left atrial volume index; LAVpre: left atrial volume index just before left atrial contraction

## Discussion

The aim of our study was to evaluate the impact of hypertension on LA structure, function, and LACI in patients with DM using CMR-FT. The results showed that after adjusting for age, sex, BSA, and smoking history, patients with DM and hypertension had a reduced total LAEF and active LAEF, as well as lower LA phasic strains, compared to those without hypertension. Moreover, LACI was found to be higher in the patients with DM and hypertension group compared to the group without hypertension. The results of multivariable analysis suggest the potential for hypertension to exert an influence on LA booster strain and LACI in patients with DM, all the while recognizing the notable co-variation between LACI and the LA volume index (LAVI) that warrants thoughtful consideration.

### The defining features of structural and functional cardiac impairment in individuals with DM

DM is associated with a range of mechanisms that can contribute to the deterioration of cardiac function. In patients with DM, early onset cardiomyopathy typically presents as diastolic dysfunction, despite preserved LVEF [19]. The focus on LV strain has been a central theme in diabetes-related cardiac research, as LV GLS is closely linked to the interstitial fibrosis and activity characteristics of the myocardium[20, 21]. DM is known to impair both LA and LV function through similar pathophysiological mechanisms [9]. In recent years, the assessment of LA function has garnered significant attention among researchers.

LA dysfunction may not always occur as a secondary consequence of LV dysfunction. Research conducted in a canine model suggest that LA dysfunction can be identified in the early stages of heart failure with preserved ejection fraction development and, as a result, can impair overall cardiac performance [22]. The results of recent studies revealed a decrease in LA reservoir and conduit strain in patients with early-diagnosed DM, even in the absence of any other comorbidities [23]. The effects of booster pump strain on patients with DM have been shown to be inconsistent in various studies, ranging from reduction to preservation or even an increase [24–27]. This variability may be attributed to the alternating compensatory and decompensatory changes in the LA pump function throughout the progression of the disease. Additionally, the Frank-Starling mechanism, which has a known effect on the LA, cannot be disregarded as a potential contributor to the observed results [28].

### The structural and functional alterations of the LA in individuals with co-occurring diabetes and hypertension

Around half of hypertension patients are insulin resistant, and this disturbance in insulin metabolism has been increasingly linked to the development of hypertension and related cardiovascular diseases (CVD) [2]. A recent examination of the Framingham data revealed that people diagnosed with both hypertension and DM had higher mortality rates and a greater risk of CVD events compared to those with only DM who had normal blood pressure [29]. Elevated pressures in the left atrium (LA) have been documented in both human patients with arterial hypertension and in animal models of the condition. Upon the onset of arterial hypertension, the left ventricle is subjected to increased afterload, leading to elevated filling pressures in the left ventricle, which may subsequently result in increased pressure within the LA [30]. Increased LA pressure causes deterioration of LA phasic strain.

The results of our study indicate that in individuals with both DM and hypertension, the phasic volume of the left atrium was increased. After adjusting for other risk factors, hypertension was found to be an independent contributor to decreased LA booster strain in patients with DM. Previous research has yielded inconsistent findings regarding changes in LA booster strain in individuals with DM. Our study provides new insight into the altered LA strain patterns in this population. The worsening of LA booster strain in individuals with hypertension may be due to a combination of more severe metabolic abnormalities, the influence of neurohumoral factors, and higher LV load, which exacerbates the process of LA pump dysfunction in individuals with both DM and hypertension [31, 32].

### Left atrioventricular coupling alterations in individuals with coexisting DM and hypertension

While the independent prognostic values of LV and LA parameters in predicting heart failure (HF) have been recognized, it is noteworthy that the interconnected physiological relationship between the LA and the LV suggests that evaluating alterations in LACI potentially offer a more comprehensive reflection of left heart dysfunction [33].

In our study involving patients with DM, elevated LACI was observed in those with concurrent hypertension, and hypertension was identified as an independent contributor to LACI. This observation suggests a potential association between elevated LACI and the presence of hypertension in patients with DM. It is noteworthy that LACI serves as a novel indicator for early identification of cardiovascular disease risk in patients without existing cardiovascular disease symptoms. The utility of LACI in patients with cardiovascular disease who display both LA and LV enlargement has yet to be established. To solidify its relevance and practical application in diverse disease scenarios, comprehensive research endeavors, including animal studies and clinical trials, are imperative.

### Limitation

Our study has provided valuable insights, but certain limitations should be acknowledged. Firstly, being a single-center study, the presence of inherent selection bias cannot be disregarded. Secondly, our observation of LACI did not account for simultaneous enlargement of the left atrium and left ventricle, however, its predictive value for adverse cardiovascular events has been established. Finally, as a retrospective study, it remains to be seen through further large-scale, multicenter prospective studies whether the decline in LA pump function and LACI alteration in patients with DM and hypertension has implications for their long-term prognosis.

## Conclusions

In patients with DM combined with hypertension, there is a reduction in LA phasic strain, with hypertension being an independent factor contributing to the decrease in LA booster strain. Furthermore, elevated LACI was observed in the hypertensive population, indicating potential atrioventricular coupling index alterations. These findings provide imaging evidence for the clinical management of this patient population.

## Data Availability

The datasets generated and/or analyzed during the current study are available from the corresponding author upon reasonable request.

## Declarations

### Founding

This work was supported by the National Natural Science Foundation of China (81771887, 81771897, 82120108015), and the 1·3·5 project for disciplines of excellence, West China Hospital, Sichuan University (ZYGD18013).

## Abbreviations

LAVImax: maximum left atrial volume index
LAVImin: minimum left atrial volume index
LAVIpre: left atrial volume index just before left atrial contraction
LAEF: left atrial emptying fraction
ε_s_: reservoir strain
ε_e_: conduit strain
ε_a_: booster strain
LVEDVI: left ventricular end-diastolic volume index
LVESVI: left ventricular end-systolic volume index
LVMI: left ventricular mass index
LVRI: left ventricular mass to end-diastolic ratio
LV GLS: left ventricular global peak longitudinal strain
LACI: left atrioventricular coupling index

## References

[1] Lin CH, Wei JN, Fan KC, Fang CT, Wu WC, Yang CY, Lin MS, Shih SR, Hua CH, Hsein YC (2022). Different cutoffs of hypertension, risk of incident diabetes and progression of insulin resistance: a prospective cohort study. J Formos Med Assoc.

[2] Jia G, Sowers JR (2021). Hypertension in diabetes: an update of Basic Mechanisms and Clinical Disease. Hypertension.

[3] McHugh K, DeVore AD, Wu J, Matsouaka RA, Fonarow GC, Heidenreich PA, Yancy CW, Green JB, Altman N, Hernandez AF (2019). Heart failure with preserved ejection fraction and diabetes: JACC state-of-the-art review. J Am Coll Cardiol.

[4] Karwi QG, Ho KL, Pherwani S, Ketema EB, Sun Q, Lopaschuk GD (2022). Concurrent diabetes and heart failure: interplay and novel therapeutic approaches. Cardiovasc Res.

[5] Nagueh SF, Khan SU. Left atrial strain for Assessment of Left ventricular diastolic function: focus on populations with normal LVEF. JACC Cardiovasc Imaging; 2022. 10.1016/j.jcmg.2022.10.011 36752445

[6] Schuster A, Backhaus SJ, Stiermaier T, Navarra JL, Uhlig J, Rommel KP, Koschalka A, Kowallick JT, Lotz J, Gutberlet M (2019). Left atrial function with MRI enables Prediction of Cardiovascular events after myocardial infarction: insights from the AIDA STEMI and TATORT NSTEMI trials. Radiology.

[7] Tadic M, Cuspidi C (2021). Left atrial function in diabetes: does it help?. Acta Diabetol.

[8] Thomas L, Marwick TH, Popescu BA, Donal E, Badano LP (2019). Left atrial structure and function, and left ventricular diastolic dysfunction: JACC state-of-the-art review. J Am Coll Cardiol.

[9] Backhaus SJ, Kowallick JT, Stiermaier T, Lange T, Koschalka A, Navarra JL, Uhlig J, Lotz J, Kutty S, Bigalke B (2020). Atrioventricular mechanical coupling and major adverse cardiac events in female patients following acute ST elevation myocardial infarction. Int J Cardiol.

[10] Germans T, Götte MJ, Nijveldt R, Spreeuwenberg MD, Beek AM, Bronzwaer JG, Visser CA, Paulus WJ, van Rossum AC (2007). Effects of aging on left atrioventricular coupling and left ventricular filling assessed using cardiac magnetic resonance imaging in healthy subjects. Am J Cardiol.

[11] Lang RM, Badano LP, Mor-Avi V, Afilalo J, Armstrong A, Ernande L, Flachskampf FA, Foster E, Goldstein SA, Kuznetsova T (2015). Recommendations for cardiac chamber quantification by echocardiography in adults: an update from the American Society of Echocardiography and the European Association of Cardiovascular Imaging. J Am Soc Echocardiogr.

[12] Truong VT, Palmer C, Wolking S, Sheets B, Young M, Ngo TNM, Taylor M, Nagueh SF, Zareba KM, Raman S et al. Normal left atrial strain and strain rate using cardiac magnetic resonance feature tracking in healthy volunteers. Eur Heart J Cardiovasc Imaging 2019. 10.1093/ehjci/jez157 31504357

[13] Pathan F, Zainal Abidin HA, Vo QH, Zhou H, D’Angelo T, Elen E, Negishi K, Puntmann VO, Marwick TH, Nagel E. Left atrial strain: a multi-modality, multi-vendor comparison study. Eur Heart J Cardiovasc Imaging 2019. 10.1093/ehjci/jez303 31848575

[14] Zhang G, Shi K, Yan WF, Li XM, Li Y, Guo YK, Yang ZG (2022). Effects of diabetes mellitus on left ventricular function and remodeling in hypertensive patients with heart failure with reduced ejection fraction: assessment with 3.0 T MRI feature tracking. Cardiovasc Diabetol.

[15] Li XM, Yan WF, Jiang L, Shi K, Ren Y, Han PL, Peng LQ, Guo YK, Yang ZG (2022). Impact of T2DM on right ventricular systolic dysfunction and interventricular interactions in patients with essential hypertension: evaluation using CMR tissue tracking. Cardiovasc Diabetol.

[16] Deng H, Hu P, Li H, Zhou H, Wu X, Yuan M, Duan X, Lao M, Wu C, Zheng M (2022). Novel lipid indicators and the risk of type 2 diabetes mellitus among chinese hypertensive patients: findings from the Guangzhou Heart Study. Cardiovasc Diabetol.

[17] Chamberlain JJ, Rhinehart AS, Shaefer CF, Neuman A (2016). Diagnosis and management of diabetes: Synopsis of the 2016 american Diabetes Association Standards of Medical Care in Diabetes. Ann Intern Med.

[18] Shi R, Shi K, Huang S, Li X, Xia CC, Li Y, He S, Li ZL, He Y, Guo YK (2022). Association between Heart failure with preserved left ventricular ejection fraction and impaired left atrial phasic function in hypertrophic cardiomyopathy: evaluation by Cardiac MRI Feature Tracking. J Magn Reson Imaging.

[19] Pezel T, Viallon M, Croisille P, Sebbag L, Bochaton T, Garot J, Lima JAC, Mewton N (2021). Imaging interstitial fibrosis, left ventricular remodeling, and function in Stage A and B Heart failure. JACC Cardiovasc Imaging.

[20] Jensen MT, Fung K, Aung N, Sanghvi MM, Chadalavada S, Paiva JM, Khanji MY, de Knegt MC, Lukaschuk E, Lee AM (2019). Changes in Cardiac morphology and function in individuals with diabetes Mellitus: the UK Biobank Cardiovascular magnetic resonance substudy. Circ Cardiovasc Imaging.

[21] Lee MMY, McMurray JJV, Lorenzo-Almorós A, Kristensen SL, Sattar N, Jhund PS, Petrie MC (2019). Diabetic cardiomyopathy. Heart.

[22] Zakeri R, Moulay G, Chai Q, Ogut O, Hussain S, Takahama H, Lu T, Wang XL, Linke WA, Lee HC et al. Left atrial remodeling and atrioventricular coupling in a Canine Model of Early Heart failure with preserved ejection fraction. Circ Heart Fail 2016, 9(10). 10.1161/CIRCHEARTFAILURE.115.003238 PMC5082983 27758811

[23] Shen MT, Guo YK, Liu X, Ren Y, Jiang L, Xie LJ, Gao Y, Zhang Y, Deng MY, Li Y (2022). Impact of BMI on left atrial strain and abnormal atrioventricular Interaction in patients with type 2 diabetes Mellitus: a Cardiac magnetic resonance feature tracking study. J Magn Reson Imaging.

[24] Mondillo S, Cameli M, Caputo ML, Lisi M, Palmerini E, Padeletti M, Ballo P (2011). Early detection of left atrial strain abnormalities by speckle-tracking in hypertensive and diabetic patients with normal left atrial size. J Am Soc Echocardiogr.

[25] Liu Y, Wang K, Su D, Cong T, Cheng Y, Zhang Y, Wu J, Sun Y, Shang Z, Liu J (2014). Noninvasive assessment of left atrial phasic function in patients with hypertension and diabetes using two-dimensional speckle tracking and volumetric parameters. Echocardiography.

[26] Tadic M, Ilic S, Cuspidi C, Ivanovic B, Bukarica L, Kostic N, Marjanovic T, Kocijancic V, Celic V (2015). Left and right atrial phasic function and deformation in untreated patients with prediabetes and type 2 diabetes mellitus. Int J Cardiovasc Imaging.

[27] Steele JM, Urbina EM, Mazur WM, Khoury PR, Nagueh SF, Tretter JT, Alsaied T (2020). Left atrial strain and diastolic function abnormalities in obese and type 2 diabetic adolescents and young adults. Cardiovasc Diabetol.

[28] Anwar AM, Geleijnse ML, Soliman OI, Nemes A, ten Cate FJ (2007). Left atrial Frank-Starling law assessed by real-time, three-dimensional echocardiographic left atrial volume changes. Heart.

[29] Chen G, McAlister FA, Walker RL, Hemmelgarn BR, Campbell NR (2011). Cardiovascular outcomes in framingham participants with diabetes: the importance of blood pressure. Hypertension.

[30] Alsharari R, Oxborough D, Lip GYH, Shantsila A (2021). Myocardial strain imaging in resistant hypertension. Curr Hypertens Rep.

[31] Koenen M, Hill MA, Cohen P, Sowers JR (2021). Obesity, adipose tissue and vascular dysfunction. Circ Res.

[32] Tynjälä A, Forsblom C, Harjutsalo V, Groop PH, Gordin D (2020). Arterial stiffness predicts mortality in individuals with type 1 diabetes. Diabetes Care.

[33] Sengupta PP, Narula J (2014). À LA mode atrioventricular mechanical coupling. JACC Cardiovasc Imaging.

